# Concentration-dependent antimicrobial effects of two mouthwashes on key periodontal pathogens: An *in vitro* study

**DOI:** 10.6026/973206300222823

**Published:** 2026-03-31

**Authors:** M.M Dayakar, Aishwarya Bruhanmath, Prakash Pai Gurpur, Shivananda K Hiranya, L.S Shilpa, Vidyalakshmi S Rao, Sarja Ishwarya

**Affiliations:** 1 Department of Periodontology, KVG Dental College and Hospital, Sullia, Karnataka, India;

**Keywords:** eriodontal pathogens, Herbal mouthwash, *Porphyromonas gingivalis*, *Aggregatibacter actinomycetemcomitans*, Antimicrobial efficacy

## Abstract

Periodontal diseases, caused by pathogenic dental plaque biofilm, are often treated with chlorhexidine, but its long-term use can lead to undesirable side effects. Therefore, it is of interest to compare the antimicrobial efficacy of two herbal mouthwashes, Oralife and Ora-T, against periodontal pathogens *Porphyromonas gingivalis* and *Aggregatibacter actinomycetemcomitans* using the disc diffusion method. Results showed that Oralife exhibited significantly larger zones of inhibition than Ora-T at all tested concentrations. No inhibitory effect was observed at the lowest concentration. Thus, we show Oralife's potential as a more effective herbal alternative for periodontal therapy.

## Background:

Periodontal diseases are chronic inflammatory conditions that primarily affect the supporting structures of the teeth, including the gums, periodontal ligament and alveolar bone [1]. These diseases are a leading cause of tooth loss in adults worldwide and can significantly impact the quality of life due to pain, discomfort and aesthetic concerns. The primary cause of periodontal disease is dental plaque, a complex biofilm that accumulates on the teeth [2]. Within this biofilm, pathogenic microorganisms, including *Porphyromonas gingivalis* and *Aggregatibacter actinomycetemcomitans*, initiate and sustain the inflammatory response. These bacteria possess virulence factors such as tissue invasiveness, immune modulation and the ability to evade host defenses, which makes them central to disease progression and tissue destruction [3]. Traditional periodontal therapy primarily involves mechanical plaque control, such as tooth brushing and professional scaling and root planing. However, these methods often fail to completely eliminate pathogens, especially from deep periodontal pockets, furcation areas and in patients with compromised oral hygiene practices [4]. To complement mechanical therapy and enhance microbial reduction, chemical plaque control agents, particularly antimicrobial mouthwashes, are widely used. These mouthwashes help control bacterial growth, reduce inflammation and promote periodontal health by targeting specific microbial species responsible for disease progression [5]. Chlorhexidine gluconate has long been regarded as the gold standard in antimicrobial mouthwashes due to its broad-spectrum activity and substantivity [6]. It is highly effective in reducing bacterial load, controlling gingivitis and supporting healing after periodontal surgery. However, the long-term use of chlorhexidine is associated with several undesirable side effects, including tooth staining, taste alterations and mucosal irritation, which can reduce patient compliance and limit its use in long-term periodontal management [7]. These limitations have prompted the exploration of alternative mouthwashes, particularly herbal and naturally derived products, which may offer comparable antimicrobial efficacy without the associated side effects [8]. Oralife and Ora-T are two commercially available herbal mouthwashes that are gaining popularity in clinical practice. Oralife is composed of botanical extracts that are known for their antibacterial, antioxidant and anti-inflammatory properties [9]. These extracts are believed to help inhibit periodontal pathogens and support overall gingival health [8]. Ora-T, on the other hand, contains ingredients such as turmeric, honey and triphala, which have been used in traditional medicine for their antimicrobial, anti-inflammatory and wound-healing properties [10]. Contemporary studies have also demonstrated the effectiveness of these ingredients against various microbial pathogens. Despite their increasing use, scientific studies directly comparing the antimicrobial efficacy of Oralife and Ora-T against periodontal pathogens are limited and their clinical utility remains underexplored [11]. *in vitro* antimicrobial testing provides a controlled, reproducible method for evaluating the potential of oral hygiene products before clinical application. The disc diffusion method is a standard technique used to assess the antimicrobial activity of agents, particularly under anaerobic conditions, which is essential for studying pathogens like *P. gingivalis* and *A. actinomycetemcomitans* that thrive in low-oxygen environments [12]. Therefore, it is of interest to report to assess the antimicrobial efficacy of Oralife and Ora-T mouthwashes against key periodontal pathogens, *P. gingivalis* and *A. actinomycetemcomitans*, using the disc diffusion method.

## Methodology:

The present study was designed as an *in vitro* experimental study to evaluate and compare the antimicrobial efficacy of Oralife and Ora-T mouthwashes against selected periodontal pathogens using the disc diffusion method under anaerobic conditions.

Using the formula,

n =(2(SD)2 (Z1-α/2)+(Zβ)2)/(d)2

Where, SD = Standard Deviation

Z1-α/2 = 1.96 AT 95% Confidence Interval

Zβ = 0.84 AT 80% power

d = Mean Difference

[1] Substituting the values, we get n =8.8 approximately equals to 9

[2] Further assuming 10% sample loss n=10

[3] Therefore, the total sample size is per group 10 per group.

**Figure 1:**
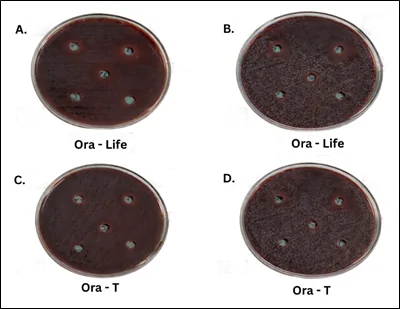
A) Agar well diffusion assay showing the antibacterial activity of Oralife against *Aggregatibacter actinomycetemcomitans* (Aa) at different concentrations. Zones of inhibition indicate concentration-dependent antimicrobial efficacy; B) Agar well diffusion assay showing the antibacterial activity of Oralife against *Porphyromonas gingivalis* (Pg) at different concentrations. Zones of inhibition indicate concentration-dependent antimicrobial efficacy; C) Agar well diffusion assay showing the antibacterial activity of Ora-T mouthwash against *Porphyromonas gingivalis* (Pg) at different concentrations. Zones of inhibition indicate concentration-dependent antimicrobial efficacy; D) Agar well diffusion assay showing the antibacterial activity of Ora-T mouthwash against *Aggregatibacter actinomycetemcomitans* (Aa) at different concentrations. Zones of inhibition indicate concentration- dependent antimicrobial efficacy;

Standard reference strains of *Porphyromonas gingivalis* ATCC 33277 and *Aggregatibacter actinomycetemcomitans* ATCC 29523 were procured and used for antimicrobial testing. The organisms were maintained and subcultured under strict anaerobic conditions to ensure viability and purity. Two commercially available mouthwashes, Oralife and Ora-T, were selected as test compounds. Dimethyl sulfoxide (DMSO) was used as the solvent for preparing stock and working solutions. Blood agar was used as the culture medium for disc diffusion testing due to its suitability for anaerobic periodontal pathogens. Fresh bacterial colonies were transferred using a sterile loop into sterile broth. The turbidity of the bacterial suspension was visually adjusted to match a 0.5 McFarland standard, corresponding to approximately 1.5 x 10^8^ CFU/ml. Standardization was performed within 15 minutes prior to inoculation to ensure uniform bacterial density across all test plates. Blood agar plates were brought to room temperature prior to inoculation. A sterile cotton swab was dipped into the standardized inoculum, rotated against the wall of the tube to remove excess fluid and used to swab the entire surface of the agar plate uniformly in three directions, rotating the plate approximately 60° between streaking. The inoculated plates were allowed to stand for 3-15 minutes to permit absorption of excess moisture. A stock solution was prepared by dissolving 10 mg of each mouthwash sample in 1 ml of DMSO. From this stock, working concentrations of 75, 50, 25, 10 and 5 µl/ml were prepared. Using a sterile heated hollow tube of 5 mm diameter, five wells were made on each agar plate. A volume of 50 µl of each concentration was dispensed into the respective wells using a micropipette. The plates were incubated within 15 minutes of compound application. Plates were inverted, stacked no more than five high and incubated at 37°C for 48-72 hours in an anaerobic jar. Following incubation, plates showing confluent or near-confluent bacterial growth were examined (Figure 1). The diameter of the zone of inhibition around each well was measured in millimeters using a calibrated measuring device. Antimicrobial activity was interpreted based on the presence or absence of inhibition zones. Statistical analysis will be performed by using the SPSS software. Descriptive will be calculated and expressed as mean and standard deviation for each variable. Analysis of the data between groups will be carried out by paired and unpaired t test and ANOVA. P < 0.05 was considered as statistically significant. We hereby confirm that *Group Pharmaceuticals* has no conflict of interest* in relation to the results of the study titled "Concentration-Dependent Antimicrobial Effect of Two Mouthwashes on key periodontal pathogens: An *in vitro* study". Group Pharmaceuticals had no role in the study design, data collection, data analysis, interpretation of results, manuscript preparation, or decision to publish. The study outcomes were generated independently by the investigators and the results were reported objectively without any influence from the sponsoring organization. This declaration is provided to ensure "transparency and ethical compliance" with journal and research publication standards. This study was financially supported by a research grant from Group pharmaceuticals. The authors declare that although the study was funded by Group pharmaceuticals the company had no role in study design, data collection, analysis, manuscript preparation or publication.

## Results:

The antimicrobial activity of Oralife and Ora-T mouthwashes against *Porphyromonas gingivalis* (Pg) ATCC 33277 and *Aggregatibacter actinomycetemcomitans* (Aa) ATCC 29523 was evaluated using the disc diffusion method at five different concentrations. The efficacy of each mouthwash was assessed by measuring the mean zone of inhibition (mm) and statistically analyzed using one-way ANOVA and unpaired t-test. For *Porphyromonas gingivalis*, Oralife demonstrated a pronounced concentration-dependent antimicrobial effect. The highest mean zone of inhibition was observed at 75 µl/ml (24 ± 1.03 mm), followed by 50 µl/ml (18 ± 2.00 mm), 25 µl/ml (16 ± 1.54 mm) and 10 µl/ml (11 ± 1.20 mm). No inhibitory effect was observed at 5 µl/ml. One-way ANOVA revealed a statistically significant difference across the concentrations tested (p < 0.001). Ora-T also showed antimicrobial activity against *P. gingivalis*, with mean inhibition zones of 15 ± 1.54 mm, 14 ± 1.85 mm and 12 ± 2.23 mm at 75, 50 and 25 µl/ml respectively, while resistance was noted at lower concentrations. ANOVA analysis indicated a statistically significant concentration-dependent effect (p < 0.001). Intergroup comparison using the unpaired t-test demonstrated that Oralife produced significantly greater inhibition zones than Ora-T at corresponding concentrations (p < 0.001). Against *Aggregatibacter actinomycetemcomitans*, Oralife exhibited notable antimicrobial activity at higher concentrations, with maximum inhibition at 75 µl/ml (21 ± 3.21 mm), followed by 50 µl/ml (16 ± 0.00 mm) and 25 µl/ml (13 ± 0.00 mm). No inhibitory zones were observed at 10 and 5 µl/ml. These differences were statistically significant on ANOVA (p < 0.001). Ora-T showed inhibition at 75 µl/ml (14 ± 3.51 mm), 50 µl/ml (13 ± 3.54 mm), 25 µl/ml (10 ± 2.15 mm) and 10 µl/ml (9 ± 2.26 mm); with resistance at 5 µl/ml. ANOVA confirmed significant differences among concentrations (p < 0.001). Unpaired t-test analysis revealed that Oralife demonstrated significantly superior antimicrobial efficacy compared to Ora-T at equivalent concentrations (p < 0.001) (Table 1). Overall, Oralife exhibited significantly greater antimicrobial activity than Ora-T against both periodontal pathogens tested.

## Discussion:

The present *in vitro* study evaluated and compared the antimicrobial efficacy of two commercially available herbal mouthwashes, Oralife and Ora-T, against key periodontal pathogens *Porphyromonas gingivalis* and *Aggregatibacter actinomycetemcomitans* using the disc diffusion method. The results demonstrated that both mouthwashes possessed measurable antimicrobial activity, with a clear concentration-dependent inhibitory effect against the tested organisms. However, Oralife consistently produced significantly larger zones of inhibition than Ora-T at corresponding concentrations against both pathogens. The superior efficacy of Oralife, particularly at higher concentrations (75 and 50 µl/ml), suggests a stronger antibacterial potential, possibly attributable to a higher concentration or synergistic action of its botanical constituents. The absence of inhibitory zones at lower concentrations for both mouthwashes indicates a threshold concentration is required to exert bactericidal or bacteriostatic effects. These findings support the rationale that herbal formulations can act as effective adjuncts in periodontal therapy, although their antimicrobial potency varies based on formulation and concentration. The findings of the present study are in agreement with earlier research demonstrating the antimicrobial efficacy of herbal mouthwashes against periodontal pathogens. Aslam *et al.* (2025) [13] reported that herbal oral rinses exhibited significant inhibitory activity against *P. gingivalis* and *A. actinomycetemcomitans*, though their efficacy was generally lower than chlorhexidine but adequate for adjunctive use in periodontal therapy. Similarly, Ardakani *et al.* (2022) [14] observed a concentration-dependent antimicrobial effect of herbal mouthwashes against anaerobic periodontal bacteria, reinforcing the importance of adequate dosing for clinical effectiveness.

Studies evaluating individual herbal components provide further insight into the present findings. Turmeric (curcumin), a key ingredient in Ora-T, has been shown to possess antimicrobial and anti-inflammatory properties against periodontal pathogens, as reported by Alalwan *et al.* (2017) [15] through its antibacterial action was moderate compared to synthetic agents. Triphala, another component of Ora-T, has demonstrated inhibitory effects against *P. gingivalis* and *A. actinomycetemcomitans* *in vitro*, as shown by More *et al.* (2008) [16] supporting its traditional use in oral health care. The superior efficacy of Oralife observed in the present study aligns with findings by Cowan and others, who suggested that polyherbal formulations may exhibit enhanced antimicrobial activity due to synergistic interactions among phytochemicals. An *in vitro* study by Nagappan *et al.* (2022) [17] reported that mouthwashes containing multiple botanical extracts showed broader and stronger antibacterial effects than single-herb formulations. Comparative studies between herbal and conventional mouthwashes have yielded similar trends. Likewise, Figueiredo *et al.* (2022) [18] emphasized that herbal formulations could reduce pathogenic bacterial load, especially when used as adjuncts to mechanical plaque control. The resistance observed at lower concentrations in the present study is consistent with reports by Prasanth *et al.* (2011) [19] who noted that sub- therapeutic concentrations of herbal rinses often fail to inhibit obligate anaerobes effectively. The strengths of this study include the use of standard ATCC strains, standardized inoculum preparation and evaluation under anaerobic conditions, which enhance the reliability and reproducibility of the results. The assessment of multiple concentrations allowed for a clear understanding of the dose-dependent antimicrobial effects. However, the *in vitro* nature of the study represents a key limitation, as it does not replicate the complex oral environment, including saliva, biofilm architecture and host immune factors. Additionally, the absence of a gold-standard control such as chlorhexidine limits direct comparison with established antimicrobial benchmarks. The findings suggest that Oralife and to a lesser extent Ora-T, may serve as effective herbal adjuncts in chemical plaque control for periodontal therapy, particularly for patients who are intolerant to chlorhexidine. However, clinical extrapolation should be approached with caution. Future research should focus on randomized controlled clinical trials to evaluate the long-term efficacy, substantivity and safety of these mouthwashes *in vivo*. Further studies exploring their effects on multispecies biofilms, cytotoxicity to oral tissues and comparative effectiveness with chlorhexidine would provide valuable clinical insight.

## Conclusion:

We show that both Oralife and Ora-T mouthwashes exhibit concentration-dependent antimicrobial activity against *Porphyromonas gingivalis* and *Aggregatibacter actinomycetemcomitans*. Oralife showed superior efficacy compared to Ora-T at equivalent concentrations. Thus, we show that herbal mouthwashes could serve as potential adjuncts in periodontal therapy, but further *in vivo* and clinical studies are needed to confirm their clinical effectiveness.

## Figures and Tables

**Table 1:** Antimicrobial activity of Oralife and Ora-T mouthwashes against periodontal pathogens (disc diffusion method)

**Organism**	**Sample**	**75 µl/ml (Mean± SD)**	**50 µl/ml (Mean± SD)**	**25 µl/ml (Mean± SD)**	**10 µl/ml (Mean± SD)**	**5 µl/ml**	**p-value [ANOVA]**
*Porphyromonas gingivalis (Pg)*	Oralife	24± 1.03	18± 2.00	16± 1.54	11± 1.20	R	<0.001*
	Ora-T	15± 1.54	14± 1.85	12± 2.23	R	R	<0.001*
p-value Unpaired T-test		<0.001*	<0.001*	<0.001*	-	-	
*Aggregatibacter actinomycetemcomitans (Aa)*	Oralife	21± 3.21	16± 0.00	13± 0.00	R	R	<0.001*
	Ora-T	14± 3.51	13± 3.54	10± 2.15	9± 2.26	R	<0.001*
p-value Unpaired T-test		<0.001*	<0.001*	<0.001*	-	-	
